# Genome-wide linkage scan for colorectal cancer susceptibility genes supports linkage to chromosome 3q

**DOI:** 10.1186/1471-2407-8-87

**Published:** 2008-04-01

**Authors:** Simone Picelli, Jana Vandrovcova, Siân Jones, Tatjana Djureinovic, Johanna Skoglund, Xiao-Lei Zhou, Victor E Velculescu, Bert Vogelstein, Annika Lindblom

**Affiliations:** 1 Department of Molecular Medicine and Surgery, Karolinska Institutet, Stockholm, Sweden; 2 Ludwig Center and Howard Hughes Medical Institute, Sidney Kimmel Comprehensive Cancer Center at Johns Hopkins, Baltimore, MD, USA

## Abstract

**Background:**

Colorectal cancer is one of the most common causes of cancer-related mortality. The disease is clinically and genetically heterogeneous though a strong hereditary component has been identified. However, only a small proportion of the inherited susceptibility can be ascribed to dominant syndromes, such as Hereditary Non-Polyposis Colorectal Cancer (HNPCC) or Familial Adenomatous Polyposis (FAP). In an attempt to identify novel colorectal cancer predisposing genes, we have performed a genome-wide linkage analysis in 30 Swedish non-FAP/non-HNPCC families with a strong family history of colorectal cancer.

**Methods:**

Statistical analysis was performed using multipoint parametric and nonparametric linkage.

**Results:**

Parametric analysis under the assumption of locus homogeneity excluded any common susceptibility regions harbouring a predisposing gene for colorectal cancer. However, several loci on chromosomes 2q, 3q, 6q, and 7q with suggestive linkage were detected in the parametric analysis under the assumption of locus heterogeneity as well as in the nonparametric analysis. Among these loci, the locus on chromosome 3q21.1-q26.2 was the most consistent finding providing positive results in both parametric and nonparametric analyses Heterogeneity LOD score (HLOD) = 1.90, alpha = 0.45, Non-Parametric LOD score (NPL) = 2.1).

**Conclusion:**

The strongest evidence of linkage was seen for the region on chromosome 3. Interestingly, the same region has recently been reported as the most significant finding in a genome-wide analysis performed with SNP arrays; thus our results independently support the finding on chromosome 3q.

## Background

Colorectal cancer is a major problem in the Western world, ranking as the second most common cause of cancer-related death with a 5% lifetime risk. One of the strongest associated risk factors for colorectal cancer is a family history of the disease. From twin studies, Lichtenstein has estimated that heritable factors account for 35% of the risk [1]. Epidemiological studies suggested that the risk of developing colorectal cancer for first-degree relatives of patients diagnosed with colorectal cancer is increased by two to four-fold [2]. Several hereditary syndromes, such as FAP and HNPCC, are known where the risk of colorectal cancer development can be as high as 100% and 80%, respectively [3]. FAP and HNPCC account for less than 5% of all colorectal cancer cases [4-6], and for the great majority of families with colorectal cancer no hereditary cause of the disease has been identified. Most of these families do not fulfil the criteria for FAP or HNPCC, but still provide empirical evidence of an increased risk of developing colorectal cancer similar to the one seen in HNPCC families [7-9]. These families are characterized by a later age of onset of the disease compared to HNPCC. A proportion of the remaining familial clustering of colorectal cancer (CRC) might be due to the involvement of low penetrance alleles [10]. Two papers recently identified a locus on 8q24.21, suggested to harbour a low risk allele that predisposes to colorectal cancer [11,12]. However, some families are expected to segregate high to moderate penetrance genes inherited in a dominant manner [13-15]. Several loci have been suggested to predispose to hereditary colorectal cancer. A region on chromosome 9q22.2-31.2 was suggested from a sib-pair analysis and has been confirmed in two linkage studies [16-18].

We have recently published a genome-wide study in 18 Swedish familial colorectal cancer families suggesting candidate loci on chromosomes 11 and 14 [19]. We now extended this study and performed a genome-wide linkage analysis in an additional set of 12 Swedish non-FAP/non-HNPCC colorectal cancer families followed by a combined analysis of data from both studies.

## Methods

### Description of families analysed

In total, 30 colorectal cancer families with 275 typed individuals were included in the study. All families were of Swedish origin and were collected through the Family Cancer Clinic at the Karolinska Hospital, Stockholm, Sweden. In all cases, the diagnosis was confirmed by medical and pathological reports and informed consent was obtained from all participants. The study was undertaken in accordance with the Swedish legislation of ethical permission (2003:460) and according to the decision in the Stockholm regional ethical committee (Dnr: 2005/566-31/1). FAP and MUTYH were excluded clinically since only one of the individuals with CRC or under surveillance had more than 4 polyps. This was a family with a dominant inheritance pattern and the APC gene was screened negative. Although none of the other families (22 with a dominant pattern of inheritance) fulfilled the criteria for screening, the APC gene had been screened in 20 of the families and the MUTYH gene in 15 of the families without any clearly pathogenic mutations found [20,21]. This protocol includes microsatellite instability (MSI) test, immunohistochemistry on selected tumours in the family, and mutation screening of the DNA mismatch repair genes *hMLH1*, *hMSH2*, *hMSH6 *or *hPMS2 *in cases fulfilling Amsterdam criteria [22], cases with MSI positive tumours, or with MSI negative tumours if any person had colorectal cancer at an age before 50. Mutation screening of mismatch repair genes was performed using direct sequencing and Multiplex Ligation-dependent Probe Amplification (MLPA). All members at increased risk were counselled and offered regular colonoscopy. Eighteen out of the 30 analysed families were included in our previous screen where they were also described [19]. The 12 new families are summarized in Table 1. In the whole data set, there were 127 persons with positive colonoscopic findings. Among these, 94 individuals had colorectal cancer (70 colon cancer and 24 rectal cancer), 76 had adenomas (range 1 to 13), and 27 individuals had hyperplastic polyps only (range 1 to 16). Twenty-nine families had individuals affected in two or more generations, and one family consisted of five affected siblings. Mean age of onset in the 30 families varied from 46.7 to 73.3 years. For association studies of allelic variants in the chromosome 3 locus we used another 190 unrelated familial colorectal cancer cases and 190 healthy controls without a family history of cancer from the same region matched for gender and age.

**Table 1:** The main characteristics of the 12 new families included in the linkage study

**Family Number**	**87**	**119**	**125**	**237**	**256**	**301**	**322**	**340**	**350**	**397**	**409**	**478**
**Number of colorectal Cancer cases**	3	5	6	5	3	3	3	4	7	3	4	4
**Number of affected**	4	8	6	5	3	3	3	4	4	5	4	5

### Genotyping

Genomic DNA was extracted from peripheral blood by standard procedures. Genotyping and a first-quality check of the additional 12 families were done at deCODE Genetics (Reykjavik, Iceland). A total of 545 fluorescently labeled microsatellite markers located on the 22 autosomes and the X chromosome with an average marker density of 7.25 cM were analyzed. Overall, 97.1% of the genotypes were successfully determined. Genetic map was used as provided by deCODE.

For fine mapping of the locus on chromosome 3 and 6 an additional set of eleven and seven markers were typed, respectively. Each marker was amplified separately according to a standard PCR protocol (conditions are available upon request). The PCR products were pooled and separated on an ABI 377 DNA sequencer. Electrophoretic data were analyzed using the Genescan^® ^and Genotyper^® ^software programs (Applied Biosystems, Foster city, CA, USA). In the combined analyses, the genetic map was constructed using the Marshfield linkage maps [23].

### Linkage analysis

All genotyping data were reanalyzed concerning the Mendelian inheritance and the relationship patterns of the families using the Pedcheck program [24]. Any markers violating Mendelian rules and with ambiguous genotypes were deleted. No individuals were excluded since only very few markers (in most cases none) were incorrect. Marker allele frequencies were calculated from all the genotyped individuals. Multipoint parametric analyses including the calculation of heterogeneity LOD scores, as well as nonparametric and haplotype analyses were computed using the SimWalk2 program version 2.83 [25].

Individuals with positive colorectal findings were coded as affected only if they had colorectal cancer or adenomas with a high degree of dysplasia. Non-related family members (spouses) were coded as unaffected and any other family members as unknown. In addition, the data for chromosome 3 and 9 obtained from combined analysis from all thirty families were recoded using recently published criteria for affected status and reanalysed using age-dependant penetrance as described by Kemp et al. [18].

An autosomal dominant mode of inheritance with the disease allele frequency of 0.0001 was used while the penetrance and phenocopy rate were assumed as 80% and 5%, respectively. In addition, single point LOD score analysis on chromosome X was performed using the Fastlink program [26].

### Direct sequencing

Database analysis revealed 340 genes (NCBI build 36) within the 65 cM interval between markers D3S1558 and D3S3592. Based on their known or hypothesized function we sequenced 20 genes in the region on chromosome 3 with a positive LOD score. Sequencing of 14 genes was done as described in Sjöblom et al. [27]. For the other 6 genes we used a slightly modified protocol as described in Liu et al. [28]. In short: primers were designed to amplify all exons including exon/intron boundaries, the 5'- and 3'-UTR regions as well as the putative promoter for some of them. Primers covering all exons were designed automatically using the ExonPrimer function implemented in the UCSC database [29] and the online Primer3 software package to cover the putative promoter sequence [30]. The coding sequence of the genes was analyzed using ABI Seqscape v2.5 software, which allows alignment of multiple samples for comparative sequence analysis with the reference sequence in the databases.

### Association studies

Genotyping of 10 SNPs on chromosome 3 was performed using TaqMan SNP Genotyping Assay (Applied Biosystems, Foster City, CA) in 190 familial colorectal cancer cases and 190 healthy controls matched to patients according to their gender and age. A standard PCR was carried out in a 384-well format, with a total reaction volume of 5 μl using 6 ng genomic DNA diluted in 2.375 μl H_2_O, 2.5 μl TaqMan Universal PCR Master Mix (Applied Biosystems, Foster City, CA) and 0.125 μl Assay. The SNP genotyping assay contains two primers for amplifying the sequence and two probes for detecting alleles. Each probe contains a reporter dye at the 5'-end; specifically, the VIC dye is linked to the allele 1 probe and the FAM dye is linked to the allele 2 probe. After the initial denaturation step at 95°C for 10 min there were 40 cycles of denaturation at 92°C for 15 sec, annealing and extending at 60°C for 1 min. The fluorescence was measured after the last PCR cycle was completed by the ABI PRISM 7900 HT instrument (Applied Biosystems, Foster City, CA) in which the fluorescence intensity of each well was read. Negative and positive controls were included in all analyses as a quality control measure. Fluorescence data files from each plate were analyzed using the SDS 2.2.1 software (Applied Biosystems, Foster City, CA). We used Haploview [31] to determine whether individual variants were in equilibrium at each locus in the population (Hardy-Weinberg equilibrium) and to check for the presence of association between single SNPs or block of several SNPs (haplotypes) and CRC.

## Results

### Genome-wide linkage analysis in the combined data set

In the combined data set of the 30 families, genome-wide linkage analysis did not reveal any locus with a statistically significant LOD score. However, genome-wide HLOD scores over 1 and NPL scores over 1.3 (p-value < 0.05) providing evidence for suggestive linkage were obtained in both parametric and nonparametric linkage analyses. Overall, four regions with p-value < 0.05 were identified. The results are summarized in table 2. In the multipoint parametric analysis the highest genome-wide HLOD score providing evidence for suggestive linkage was obtained for the marker D3S1279 located at 168.28 cM (HLOD = 1.90, α = 0.45). The highest NPL score of 2.1 (p-value = 0.008) was obtained for the same marker. Three other loci, on chromosomes 2q, 6q and 7q, provided parametric HLOD higher than or equal to 1. The second most significant HLOD was reached for the region on chromosome 7q (HLOD = 1.70, α = 0.70), while considering the NPL was on chromosome 6 (NPL = 1.50, p-value = 0.03).

**Table 2:** 

**Location**	**Marker**	**HLOD**	**α-value**	**NPL Score**	**p-value**
**2q37.3**	D2S140	1.09	0.70	1.00	0.100
**3q25.1**	D3S1279	1.90	0.45	2.10	0.008
**6q23.2**	D6S270	1.30	0.40	1.50	0.030
**7q11.21**	D7S2429	1.70	0.70	1.20	0.050

Summary of all linkage peaks with HLOD scores greater than 1 and their corresponding NPL scores obtained in linkage analysis in 30 colorectal cancer families with 275 typed individuals

### Fine mapping and sequencing on chromosome 3q

**Figure 1:**
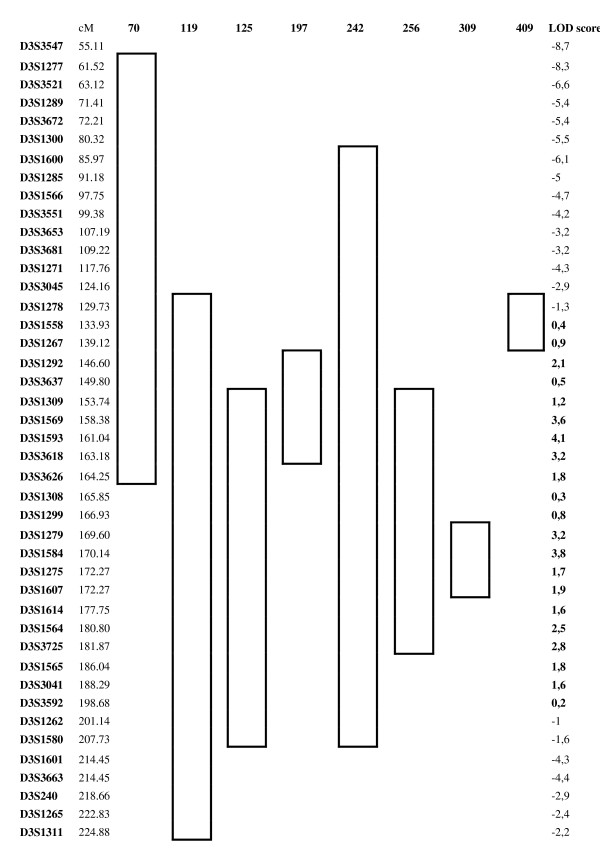
**Linkage analysis and haplotype analysis on chromosome 3 after fine mapping in the 8 linked families**. The genomic regions included in the bolded boxes indicate a positive LOD scores for those markers. The marker map is based on the Marshfield map.

Eight families with possible linkage to chromosome 3q were included in subsequent fine mapping. Positive LOD scores were detected for the region between D3S1558 and D3S3592 spanning 65 cM (Figure 1). However, no single overlapping region shared by all families was identified in the haplotype analysis. Instead, two linkage peaks were observed; one around marker D3S1593 and a second one around marker D3S1584 where six (Fam. 70, Fam. 119, Fam. 125, Fam. 197, Fam. 242, Fam. 256) and five families (Fam. 119, Fam. 125, Fam. 242, Fam. 256, Fam. 309) contributed with positive LOD scores, respectively.

In order to further investigate the linked region we sequenced 20 candidate genes (PIK3R4, ASTE1, NEK11, NUDT16, MRPL3, TOPBP1, SRPRB, RAB6B, RYK, PPP2R3A, PIK3CB, RNF7, TFDP2, SLC9A9, MGC33365, PLOD2, PLSCR1, PLSCR2, PLSCR4, PLSCR5), on the basis of their known or predicted function and position near the markers that showed the highest LOD score. We sequenced the exons, the intron/exon boundaries, 5' and 3' UTR and, for some genes, the promoter region. No deleterious mutations were found, but instead many variants (missense, silent, intronic and in the promoter), already reported in the databases, were recorded. We were particularly interested in family 242 because it gives the largest contribution to the LOD score values on chromosome 3. Ten SNPs (rs9864242, rs3738000, rs9843898, rs3762802, rs3762803, rs2291382, rs17301766, rs3192149, rs9814557, rs17197552) were present on the disease haplotype in family 242. Association studies were performed to test these 10 SNPs using 190 familial colorectal cancer cases and 190 controls without a family history. Eight SNPs had a frequency above 5%, were tested and did not show significant differences between the two groups; 2 SNPs (rs2291382 and rs3762802) had a frequency below 5%, 8 cases and 7 controls were found in the two groups respectively and no further tests were performed.

### Genome-wide linkage analysis in the 12 new families

In the 12 new families, 545 microsatellite markers were used for genotyping, compared to 400 markers in our first screening with 18 families. For this reason we analyzed the 12 new families separately as well. The strongest evidence of linkage was found on chromosome 6q22.3-24.3 with an NPL score of 2.06 (p-value = 0.01) for D6S1656. Furthermore, a p-value < 0.05 was obtained for three additional loci on chromosomes 1p36.22, 3q24-25.1, and 13q33.2. Under the assumption of locus heterogeneity, linkage was most evident in the region on chromosome 6q22-23. The highest HLOD score was obtained for marker D6S270 (HLOD = 1.68 for α = 0.4). Positive HLOD scores of 1.59 (α = 0.4) and 1.39 (α = 0.45) were seen in this analysis for both flanking markers, D6S1656 and D6S1009, respectively and supported the results obtained for this region in the nonparametric analysis. Out of the 12 families, seven provided positive LOD scores for this region. Fine mapping on chromosome 6q using additional 11 markers confirmed the results and determined the region of linkage between markers D6S1712 and D6S1637 (30 cM) with a NPL peak of 2.3 for 2 closely located markers (250 kbp), D6S976 and D6S270. No common shared haplotype was found among the linked families.

## Discussion

We have recently presented results from a genome-wide screen in 18 Swedish colorectal cancer families. In order to further evaluate and narrow down regions of suggestive linkage, we extended our family material with 12 additional families, and combined the results from the new genome-wide analysis in the 12 families with our previously published findings.

In our combined analysis containing 275 subjects from 30 non-FAP/non-HNPCC colorectal cancer families, we did not find any support for the two loci on chromosomes 11 and 14 suggested from the previous analysis of 18 of the families. Instead, we now found suggestive linkage to chromosome 3q, and the same region was also suggested from the nonparametric analysis of 12 families alone. Interestingly, our candidate region of approximately 65 cM between markers D3S1558 and D3S3592 on chromosome 3q13.31-q27.1 overlaps that recently identified as the most significant finding by Kemp et al. [32]. Their region was 17.8 Mbp in length and includes over 90 RefSeq genes. They screened two genes, representing candidates on the basis of their biology, without finding any potential pathogenic change [32]. Our region is wider and contains 340 genes; we studied 20 of them based on their known or presumptive function, or location in vicinity of the highest LOD score values, without finding any clearly pathogenic mutations.

In the analysis using the 12 new families alone, the strongest linkage (NPL = 2.3) was observed for the markers D6S976 and D6S270, and the candidate region was identified between D6S1712 and D6S1637 (30 cM). This locus was further supported in the pooled analysis using all 30 families where suggestive NPL scores for these markers were provided in nonparametric analysis. The same locus has previously been suggested from the linkage analysis using sib pairs affected with colorectal adenomas and carcinomas [17]. In that study, a smaller region on chromosome 6q23.1-23.3 located between markers D6S1040 and D6S1009 provided a significant result (p = 0.01) in a group of concordantly affected sib pairs. The region, however, failed to reach statistical significance among discordantly affected siblings and concordantly unaffected siblings [17].

No confirmation of the previously published region on chromosome 9q22.2-q31 was found. Neither was any support of linkage found when the analysis was performed using a criteria for affected status and age dependant penetrance as described by Kemp et al [18]. In the two previously published studies identifying linkage to chromosome 9q, the affected individuals had age of onset below 65 and 55 years, respectively. We also performed analysis based on age subgroups, but no linkage to chromosome 9 was detected in either subgroup.

## Conclusion

Our data support the idea of a genetic heterogeneity among colorectal cancer families, and indicate that a further subdivision of families into groups sharing similar phenotypic and molecular features is needed. Several loci with suggestive linkage were detected in the parametric analysis performed under the assumption of locus heterogeneity as well as in the nonparametric analysis. The strongest evidence of linkage was seen to the region on chromosome 3. Interestingly, the same region has recently been reported as the most significant finding in the genome-wide analysis performed with SNP arrays by Kemp et al[32]; thus, our results represent an independent confirmation, but further studies are needed in order to determine the significance of this region as well as the other regions suggested from this study.

## Competing interests

The author(s) declare that they have no competing interests.

## Authors' contributions

SP carried out the sequencing of the genes, performed the association studies, participated in the linkage analysis and drafted the manuscript. JV performed the linkage analysis and drafted the manuscript. SJ participated in the sequencing of the genes. TD participated in the linkage analysis. JS participated in the linkage analysis. XLZ participated in the linkage analysis. VV contributed to the study design. BV contributed to the study design. AL conceived the study, participated in its design and coordination and drafted the manuscript. All authors read and approved the final manuscript.

## Pre-publication history

The pre-publication history for this paper can be accessed here:


